# A multi‐omics study to monitor senescence‐associated secretory phenotypes of Alzheimer's disease

**DOI:** 10.1002/acn3.52047

**Published:** 2024-04-11

**Authors:** Jingzhi Yang, Yinge Zhou, Tianjiao Wang, Na Li, Yufan Chao, Songyan Gao, Qun Zhang, Shuo Wu, Liang Zhao, Xin Dong

**Affiliations:** ^1^ Institute of Translational Medicine Shanghai University Shanghai 200444 China; ^2^ School of Medicine Shanghai University Shanghai 200444 China; ^3^ Department of Internal Medicine Shanghai Baoshan Elderly Nursing Hospital Shanghai 200435 China; ^4^ Neurology Department Shanghai Baoshan Luodian Hospital Shanghai 201908 China; ^5^ Department of Pharmacy Shanghai Baoshan Luodian Hospital Shanghai 201908 China; ^6^ Suzhou Innovation Center of Shanghai University Suzhou 215000 Jiangsu China

## Abstract

**Objective:**

Alzheimer's disease (AD) is characterized by the progressive degeneration and damage of neurons in the brain. However, developing an accurate diagnostic assay using blood samples remains a challenge in clinic practice. The aim of this study was to explore senescence‐associated secretory phenotypes (SASPs) in peripheral blood using mass spectrometry based multi‐omics approach and to establish diagnostic assays for AD.

**Methods:**

This retrospective study included 88 participants, consisting of 29 AD patients and 59 cognitively normal (CN) individuals. Plasma and serum samples were examined using high‐resolution mass spectrometry to identify proteomic and metabolomic profiles. Receiver operating characteristic (ROC) analysis was employed to screen biomarkers with diagnostic potential. K‐nearest neighbors (KNN) algorithm was utilized to construct a multi‐dimensional model for distinguishing AD from CN.

**Results:**

Proteomics analysis revealed upregulation of five plasma proteins in AD, including RNA helicase aquarius (AQR), zinc finger protein 587B (ZNF587B), C‐reactive protein (CRP), fibronectin (FN1), and serum amyloid A‐1 protein (SAA1), indicating their potential for AD classification. Interestingly, KNN‐based three‐dimensional model, comprising AQR, ZNF587B, and CRP, demonstrated its high accuracy in AD recognition, with evaluation possibilities of 0.941, 1.000, and 1.000 for the training, testing, and validation datasets, respectively. Besides, metabolomics analysis suggested elevated levels of serum phenylacetylglutamine (PAGIn) in AD.

**Interpretation:**

The multi‐omics outcomes highlighted the significance of the SASPs, specifically AQR, ZNF587B, CRP, and PAGIn, in terms of their potential for diagnosing AD and suggested neuronal aging‐associated pathophysiology.

## Introduction

Alzheimer's disease (AD) is a neurodegenerative disorder that primarily affects the brain, one of the main characteristics of AD, is the gradual degeneration and damage of neurons within the brain.^1^ The limitations of current targeted therapeutic strategies in effectively slowing down cognitive decline in Alzheimer's disease have raised concerns. To address these concerns and improve therapeutic outcomes, it is essential to analyze and understand the molecular and cellular components of the Alzheimer's disease brain at both asymptomatic and symptomatic stages of disease progression.

It is well‐known that aging is a significant risk factor for the development of AD. Emerging evidence indicates that the number of cells expressing biomarkers of cellular senescence increases in tissues with aging, which implies that cellular senescence is an important player in organismal aging.^2^ Senescent cells progressively accumulate in aging tissues, contributing to the development of age‐related diseases through their secretion of inflammatory factors.^3^ Recent evidence suggests that the increased inflammatory response observed in AD could be influenced, at least in part, by an aggravation of cellular senescence triggered by amyloid‐β and/or tau pathology. However, the exact reasons for this association are still not fully understood.^4^ Senescent cells express senescence‐associated secretory phenotypes (SASPs), leading to the increased secretion of various proteins and metabolites, including inflammatory cytokines, chemokines, growth factors, matrix metalloproteinases, and lipids into the surrounding extracellular fluid.^5^ Thus, monitoring SASP factors shall offer a promising and feasible approach to evaluate the senescence status of aging or dementia cohort. Recent research indicates that experts consider proteomic and metabolic approaches essential for developing senescence‐related biomarkers for future therapeutic interventions aimed at targeting senescent cells.^6^

Over the past few decades, there have been numerous attempts to understand the mechanisms of aging through omics approaches. High‐resolution mass spectrometry (HRMS) has provided opportunities to quantify aging beyond using a limited number of well‐studied and selective biomarkers.^7^, ^8^ This approach can reveal age‐related changes at early stages when traditional biomarkers are not yet detectable.^9^ Recent research studies highlight the potential of proteomics and metabolomics in AD.^10^, ^11^ Proteomics involves protein digestion followed by mass spectrometry analysis to identify various proteins based on peptide sequences.^10^, ^12^ Mass spectrometry‐based proteomics in AD provides insights into protein networks related to amyloid and tau pathways, as well as other key processes like RNA splicing, lipid metabolism, and synaptic function.^13^ Metabolomics aims to identify and quantify small‐molecule metabolites in samples, revealing disruptions in sphingolipid metabolism in early stages of AD.^14^ Nevertheless, the utilization of single omics approaches yields merely a fragmented comprehension of the biological system, thereby susceptible to underlying biases in interpretation. Consequently, the integration of proteomics and metabolomics data provides a more comprehensive understanding of neuronal biology, potentially leading to the discovery of novel biomarkers for AD diagnosis.

Blood biomarkers play a crucial role in not only categorizing patients but also identifying diseases and monitoring disease progression. They significantly contribute to the decision‐making process regarding effective treatment strategies. Our previous metabolomics study with plasma samples from AD patients revealed a significant upregulation of phenylacetylglutamine (PAGIn), a gut–microbiota‐derived metabolite. We also observed a positive correlation between PAGIn and the Aβ42/Aβ40 ratio, suggesting its involvement in one or more pathophysiological mechanisms underlying AD.^15^ Ongoing research aims to identify the senescence‐associated secretory molecules that shall disclose the underlying biological processes of AD through a comprehensive omics approach. Moreover, the analysis of blood samples is an extensively utilized diagnostic procedure across multiple medical disciplines. This approach could enable deep insights into characterizing the blood proteome and metabolome and shall greatly benefit biological research.

As the field of molecular science has witnessed a paradigm shift with the advent of machine learning algorithms, facilitating data‐driven scientific exploration. The ongoing research focuses on extracting meaningful MS features and discriminating between samples based on these features.^16^ Therefore, it is necessary to utilize a machine learning‐based approach to assess the stability and predictive value of the resulting list of biomarkers. In a recent study, a machine learning classification algorithm, specifically the K‐nearest neighbor (KNN) algorithm, has been implemented to classify and detect the main four early stages of AD: non‐dementia or normal, very mild dementia, mild dementia, and moderate dementia. This study leveraged the Kaggle dataset and achieved promising results in terms of accuracy and discrimination sensitivity.^17^

We performed a comprehensive analysis of the proteome and metabolome using blood samples from individuals with AD and cognitively normal (CN). Machine learning‐based KNN algorithm was introduced to extract the MS features with significance, enhance the accuracy of model in a multi‐dimensional fashion and also promote novel strategies for AD diagnosis. This analysis not only motivated us to visit the multi‐omics phenotype associated with AD and explore the aging related SASP molecules that may elucidate the underlying pathogeneses of AD.

## Materials and Methods

### Participants recruitment and blood sample collection

Human plasma and serum specimens were collected under the auspices of Shanghai Baoshan Luodian Hospital. The study was approved by the local research ethics committee (Approval document number: No. LDYY‐KY‐2020‐04). Informed consent was obtained from all research participants, either through written consent or verbal agreement, ensuring their understanding and voluntary participation in the study. Basic demographic data were documented along with Alzheimer's Disease Assessment Scale‐Cognitive (ADAS‐Cog) Subscale. Scores on the ADAS‐Cog scale span from 0 to 75, where a score equal to or exceeding 18 signifies recognition impairment, qualifying individuals for inclusion in the Alzheimer's disease (AD) group. Conversely, a score below 18 signifies normal recognition ability and qualifies participants for inclusion in the cognitively normal (CN) group. Based on the wills of the control volunteers, some were willing to provide either plasma or serum samples. Then, collections of control plasma and control serum were performed separately. Consequently, two separate enrollment sessions were conducted: plasma samples were collected from the CN1 group, while serum samples were obtained from the CN2 group.

Collected samples were frozen and stored at −80°C in aliquots of polyethylene tubes until use. As a quality control measure, samples from each participant were pooled to create quality control samples. These samples were utilized to evaluate downstream sample preparation and MS measurements' stability, ensuring accurate and reliable results. Overall, this study was conducted in accordance with the ethical standards laid down in the 1964 Declaration of Helsinki and its later amendments, promoting the ethical conduct of research and safeguarding the welfare of the participants.

### Plasma protein extraction and digestion

Protein quantification used the Bicinchoninic Acid (BCA) assay (Beyotime, P0012). To preserve the integrity of low‐abundance proteins, we chose not to deplete high‐abundance proteins during sample preparation. Each sample had 100 μg of plasma protein diluted with 8 M urea and 0.1% FA to a final volume of 90 μL. The pH was adjusted to approximately 8.0 with 10 μL of 1 M triethylammonium bicarbonate (TEAB) buffer (Sigma, T7408). To reduce protein disulfide bonds, 2 μL of 0.5 M tris(2‐carboxyethyl) phosphine (TCEP) reagent (Thermo, 77720) was added and incubated at 500 rpm and 37°C for 1 h. After cooling, 4 μL of 1 M chloroacetamide (CAA) was mixed for 40 min. Proteins were precipitated with 700 μL of pre‐cooled acetone at −20°C for at least 4 h, then dissolved and washed with pre‐cooled 90% acetone twice. After evaporating residual acetone, proteins were reconstituted with 100 μL of 100 mM TEAB buffer. Trypsin (Promega, V5113) was added and incubated for 16 h at 37°C. The resulting peptide mixture was desalted, dried, and redissolved in 0.1% FA. Final concentration of 500 ng/μL was achieved.

### Plasma proteomics analysis

Peptide concentration was determined with a Nanodrop spectrophotometer (Thermo Fisher Scientific). For each sample, 1 μg of peptide was injected. The LC–MS instrumentation used was an EASY‐nLC 1200 coupled to a Q Exactive 480 Orbitrap mass spectrometer (Thermo Fisher Scientific, USA). Purified peptides were separated on nano HPLC‐columns (Acclaim PepMapTM RSLC, 75 μm × 25 cm, nanoViper, Thermo Scientific, USA) and eluted using a gradient of buffer A and buffer B over 60 min. MS data acquisition followed the Top10 data‐dependent MS/MS scan method. Target values for full‐scan MS spectra were set at 3 × 10^6^ in the range of 400‐1650 m/z. Fragmentation of precursor ions was achieved through higher energy C‐trap dissociation. MS/MS scans were performed at a resolution of 15,000 at m/z 200. A control step was conducted using a 500 ng Hela Protein Digest standard from ThermoScientific to assess measurement quality.

### Database search of proteins

Proteomics MS data were acquired and processed using the Xcalibur data visualization platform and Proteome Discover 2.5, both from Thermo Scientific. To identify peptides, extensive searches were performed against the protein database of Homo sapiens (sp_canonical, TAXID = 9606) obtained from the UniProt FASTA database. A stringent false discovery rate (FDR) of 0.01 was applied at both the protein and peptide levels to ensure rigorous quality control. Peptides with a minimum length of 6 amino acids were considered, and FDR estimation was performed using a decoy database. Enzymatic specificity was defined, with trypsin selected as the protease. Search parameters allowed for up to two missed cleavages and had specified precursor and fragment mass deviations. These parameters ensured accurate and reliable peptide and protein identification in the proteomics analysis.

### AD diagnostic model based on plasma proteomics

ROC curves can be used to evaluate the diagnostic or predictive capabilities of screening tests or differential proteins for Alzheimer's disease. In transfer learning, the proteomics mass spectrometry dataset was partitioned into training, testing, and validation sets following Shen's method for small sample sizes. The training group comprised 52% of the data, the testing group 17%, and the validation group 31%.^18^ To enhance accuracy, the KNN algorithm was utilized with a logarithmic transformation applied to normalize variables.^19^ KNN analysis was performed using the SPSS 18.0 Modeler platform (SPSS Software Inc., USA) and a minimum k of 3 and maximum of 5. Distances were computed with Euclidean measurement, utilizing a 10‐fold cross‐validation technique. Probabilities for targeted biomarkers represented the constructed models.

### Serum metabolomics analysis and relative response calculation

The metabolomic analysis followed established protocols as previously reported.^15^ Serum metabolites were extracted and analyzed using a UPLC‐MS system (Dionex Ultimate 3000, Q‐Exactive Plus, Thermo Scientific). Thermo Compound Discover 3.1 (Thermo Scientific, USA) was utilized for MS feature extraction and compound identification. 2‐Chloro‐L‐phenylalanine served as the internal standard for MS stability evaluation. Semiquantification was conducted by determining the relative responses (Rel. Res.) of the designated compounds. Additionally, the calculated Rel. Res. values were used to examine the significant differences between the AD and CN groups.

### Statistical analysis and graphics

The statistical analysis involved the use of Mann–Whitney *U*‐tests to compare the quantitative variables between the AD and CN groups. The up or down‐regulation of 28 plasma protein biomarkers was visualized using boxplots. The cross‐sectional correlation between molecules and MS intensities was assessed using Spearman's correlation analysis. Logistic regression models were used to explore the cross‐sectional association between molecules with statistical significance. Graphical representations were performed using GraphPad Prism 9 (GraphPad Software Inc., USA). KNN analysis was performed using IBM SPSS Statistics Modeler 18.0 (SPSS Software Inc., USA), while nonlinear surface fitting models were constructed using Origin 2021 (OriginLab Corporation, USA).

## Results

### Demographic characteristics of AD patients and cognitively controls

The assessment scores of ADAS‐Cog Subscale demonstrated that the average evaluation scores were 72.7 ± 5.6 for the AD group (*N* = 29), 6.5 ± 6.5 for the CN1 group (*N* = 29), and 6.2 ± 6.3 for the CN2 group (*N* = 30). The average age for the AD group was 70.5 ± 5.1 years, for the CN1 group was 70.7 ± 5.1 years, and for the CN2 group was 70.7 ± 5.1 years. Additionally, among the three study groups, 62% of females were in the AD group, 59% of females were in the CN1 group, and 60% of females were in the CN2 group. The average duration of education was 4.2 ± 2.4 years in the AD group, 5.6 ± 3.6 years in the CN1 group, and 5.7 ± 3.4 years in the CN2 group. Statistical analysis indicated no significant differences in age distribution and duration of education between each pair of groups.

In a previous study, metabolomics analysis was performed on plasma samples from the AD and CN1 groups, while ELISA was used to measure the levels of Aβ42, Aβ40, and T‐tau in plasma to distinguish between the groups.^15^ In this work, a proteomics analysis was performed on plasma samples of the AD and CN1 groups, and a metabolomics analysis was conducted on serum samples of the AD and CN2 groups. Detailed information and values were summarized in Table 1.

**Table 1:** Demographic characteristics of participants and layout of multi‐omics experiments.

Characteristics	AD	CN 1	CN 2
Number (*N*)	29	29	30
ADAS‐Cog score (Mean ± SD)	72.7 ± 5.6	6.5 ± 6.5	6.2 ± 6.3
Age (y)	70.5 ± 5.1	70.7 ± 5.1	70.7 ± 5.1
Education (y)	4.2 ± 2.4	5.6 ± 3.6	5.7 ± 3.4
Gender (female/male, percentage)	18/11, 62%	17/12, 59%	18/12, 60%
Aβ 40 (pg/mL)^1^	333.91 ± 94.16	303.07 ± 114.86	n.a.
Aβ 42 (pg/mL)^1^	9.42 ± 11.43	3.73 ± 2.72	n.a.
T‐tau (pg/mL)^1^	1199.26 ± 959.95	2217.14 ± 975.05	n.a.
Collected Specimen	Plasma and serum	Plasma	Serum
Omics experiment	Metabolomics	n.a.	Metabolomics
	Proteomics	Proteomics	n.a.

n.a., not applicable.

^1^ The levels of Aβ40, Aβ42, and T‐tau in plasma samples were determined using ELISA, as reported in a previous study.^15^

### Plasma proteome profiling by LC–MS/MS

**Figure 1:**
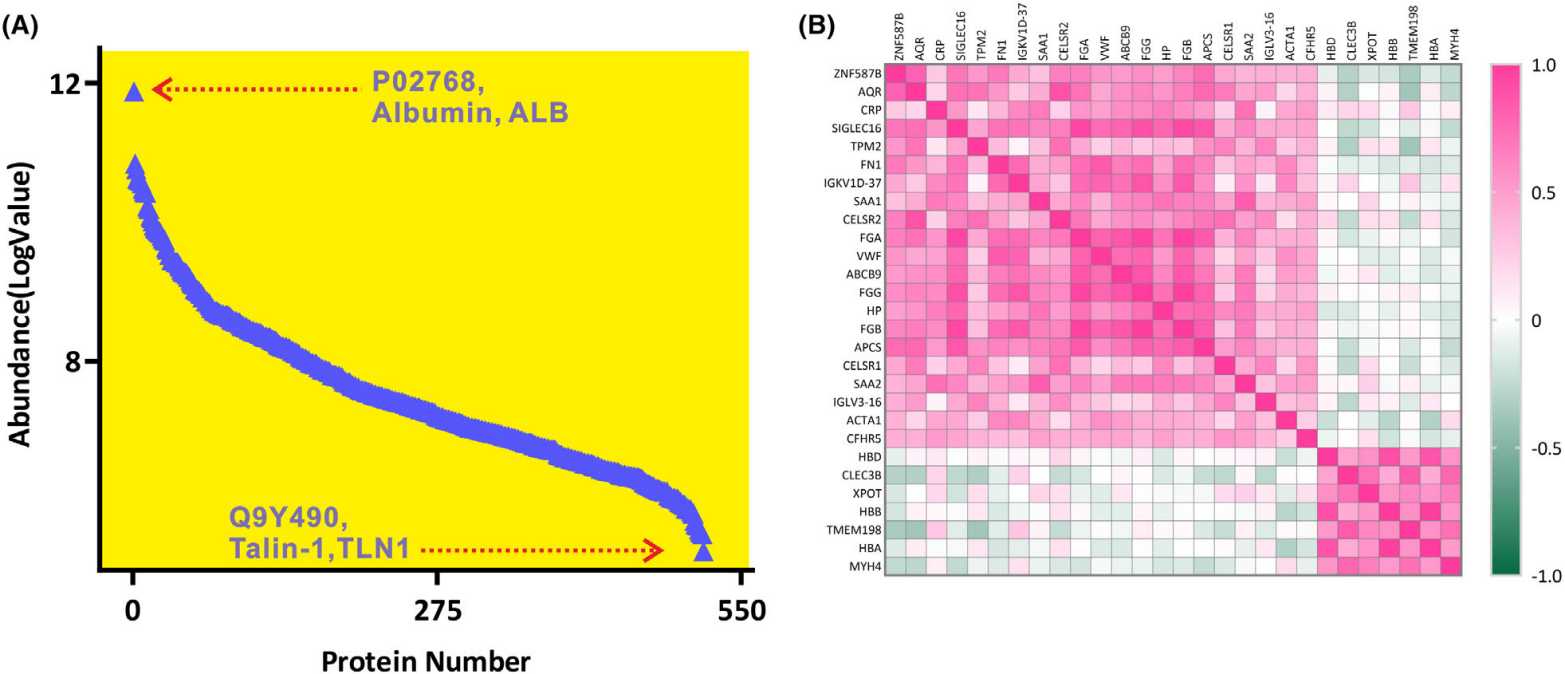
(A) Plasma proteomic profile of Alzheimer's disease approached by high‐resolution mass spectrometer, 516 non‐depleted plasma protein were identified and plotted with Log value. The most abundant protein was identified as albumin (ALB) with UniProt ID of P02768, the least abundant protein was confirmed as talin‐1 (TLN 1) with UniProt ID of Q9Y490; (B) Correlation heatmap with 28 differential proteins demonstrated their regulatory trends between AD and CN.

The raw files containing proteomics data were collected once the MS acquisition was completed. Proteome Discoverer 2.5 software (PD 2.5, Thermo Scientific, USA) was utilized to process peptide signals, annotate unique peptides to their corresponding proteins, and provide relative responses for each identified protein. Data‐dependent analysis (DDA) enabled the identification and annotation of 516 non‐depleted plasma proteins through database searching. An overview of the proteomics profile of Alzheimer's disease (AD) was depicted in Figure 1A, showcasing the average intensities of log‐transformed plasma proteins (*N* = 516). In this analysis, the most abundant protein in plasma was albumin (ALB), which had a log value of MS abundance of 11.87. Conversely, the protein with the lowest log value of MS abundance was Talin‐1 (TLN 1), which had a value of 5.27.

### Data process and differentially expressed proteins analysis

All proteomics data underwent normalization using QC samples as part of the quantitation workflow in PD 2.5, resulting in the generation of normalized MS intensities for subsequent analysis (Table S1). Out of the total annotated proteins, 334 proteins were successfully quantified with missing values below 20%, meeting the criteria for differential analysis. Subsequently, 198 proteins were selected based on the sample/control abundance ratio cutoff, with 136 proteins having ratios above 1.2 and 62 proteins having ratios below 0.8, signaling their inclusion for further analysis. The Mann–Whitney *U*‐test identified 28 candidate proteins that demonstrated significant differential expression between CN1 and AD (Table 2). Correlation heatmaps of plasma proteins illustrated the regulatory trends of the screened molecules, as depicted in Figure 1B. Boxplots revealed that 21 proteins were significantly upregulated (*p* < 0.05), while 7 proteins demonstrated significant downregulation (*p* < 0.05) in individuals with AD (Fig. S1).

**Table 2:** Differential proteins selected based on Proteome Discoverer 2.5 software.

No	UniProt accession ID	Protein name	Gene	Abundance ratio: (AD) /(CN)	Abundance ratio adjusted *p*‐value: (sample)/(control)	Subcellular location^1^
1	E7ETH6	Zinc finger protein 587B	ZNF587B	8.432	1.17194E‐07	Nucleus
2	O60306	RNA helicase aquarius	AQR	4.308	0.001005392	Nucleus
3	P02741	C‐reactive protein	CRP	3.541	0.006993768	Secreted
4	A6NMB1	Sialic acid‐binding Ig‐like lectin 16	SIGLEC16	3.181	0.017279823	Membrane
5	P07951	Tropomyosin beta chain	TPM2	2.981	0.029521031	Cytoplasm, cytoskeleton
6	P02751	Fibronectin	FN1	2.931	0.033538641	Secreted
7	P0DSN7	Probable nonfunctional immunoglobulin kappa variable 1D‐37	IGKV1D‐37	2.881	0.038515406	Cell membrane, secreted
8	P0DJI8	Serum amyloid A‐1 protein	SAA1	2.861	0.040472261	Secreted
9	Q9HCU4	Cadherin EGF LAG seven‐pass G‐type receptor 2	CELSR2	2.743	0.053606708	Cell membrane
10	P02671	Fibrinogen alpha chain	FGA	2.374	0.121653905	Secreted
11	P04275	von Willebrand factor	VWF	2.353	0.128040944	Secreted
12	Q9NP78	ABC‐type oligopeptide transporter ABCB9	ABCB9	2.353	0.128040944	Secreted
13	P02679	Fibrinogen gamma chain	FGG	2.347	0.129695671	Secreted
14	P00738	Haptoglobin	HP	2.321	0.139085688	Secreted
15	P02675	Fibrinogen beta chain	FGB	2.273	0.155309958	Secreted
16	P02743	Serum amyloid P‐component	APCS	2.257	0.161858393	Secreted
17	Q9NYQ6	Cadherin EGF LAG seven‐pass G‐type receptor 1	CELSR1	2.17	0.200901633	Cell membrane
18	P0DJI9	Serum amyloid A‐2 protein	SAA2	2.164	0.203510016	Secreted
19	A0A075B6K0	Immunoglobulin lambda variable 3–16	IGLV3‐16	2.148	0.211228324	Cell membrane, secreted
20	P68133	Actin, alpha skeletal muscle	ACTA1	2.077	0.241212237	Cytoplasm, cytoskeleton
21	Q9BXR6	Complement factor H‐related protein 5	CFHR5	2.023	0.265142733	Secreted
22	P02042	Hemoglobin subunit delta	HBD	0.546	0.237743501	Blood microparticle
23	P05452	Tetranectin	CLEC3B	0.51	0.163347401	Secreted
24	O43592	Exportin‐T	XPOT	0.46	0.09761194	Nucleus, Cytoplasm
25	P68871	Hemoglobin subunit beta	HBB	0.444	0.080222037	Blood microparticle
26	Q66K66	Transmembrane protein 198	TMEM198	0.421	0.05588057	Membrane
27	P69905	Hemoglobin subunit alpha	HBA1; HBA2	0.365	0.018902331	Blood microparticle
28	Q9Y623	Myosin‐4	MYH4	0.333	0.008978847	Cytoplasm

^1^ The subcellular location information was cited from UniProt (https://www.uniprot.org/).

### Diagnostic power of single plasma protein

**Figure 2:**
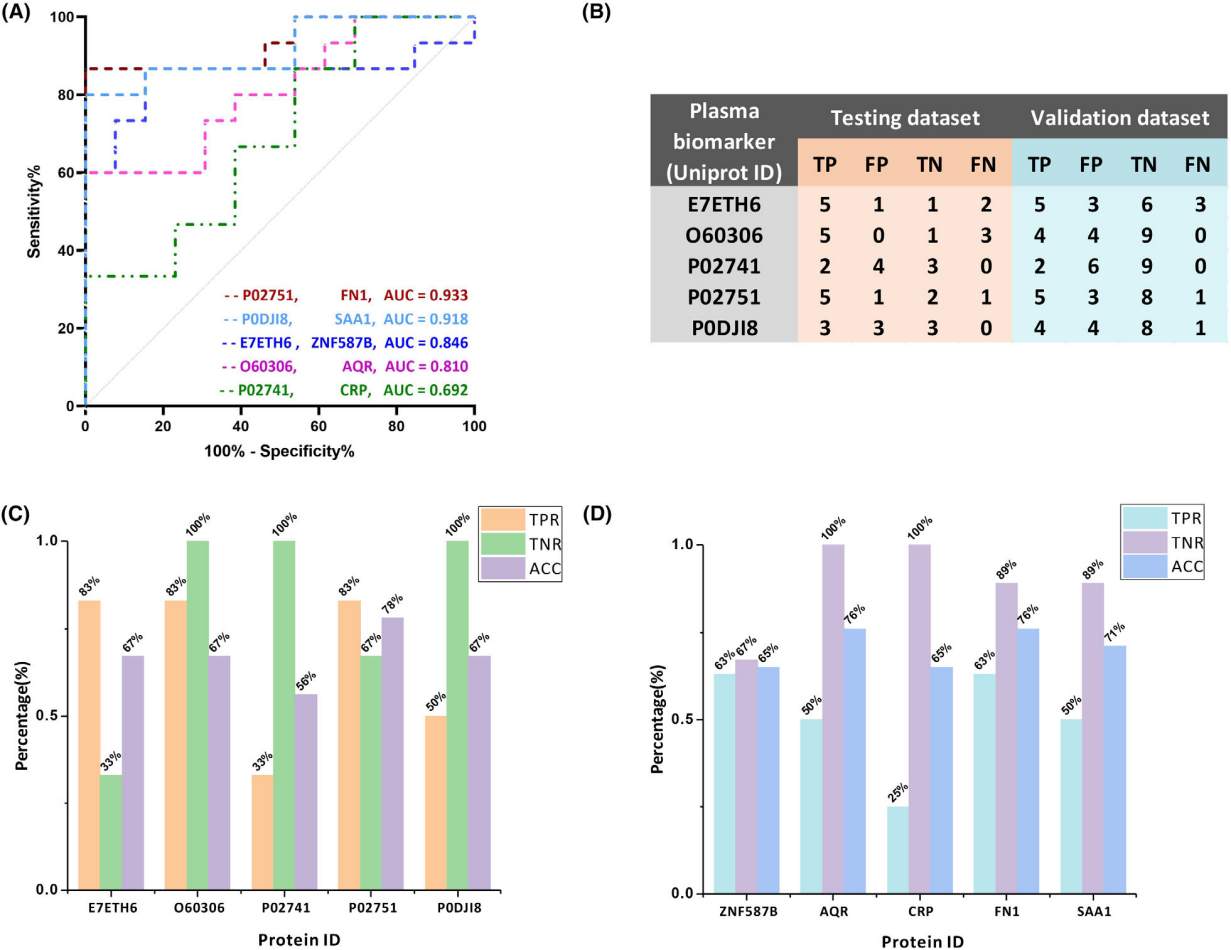
(A) ROC curves were used to calculate AUC values for five potential plasma proteins in the training dataset. FN1 had the highest AUC value (0.933), followed by SAA1 (0.918), ZNF587B (0.846), AQR (0.810), and CRP (0.692); (B) The model's evaluation on the testing and validation datasets included metrics such as true‐positive (TP), false‐positive (FP), true‐negative (TN), and false‐negative (FN), which were obtained from the training dataset using the Youden Index and cutoff values; (C) Calculated TPR, TNR, and ACC in testing dataset; (D) Calculated TPR, TNR, and ACC in validation dataset.

Twenty‐eight proteins out of 516 in a table were identified based on AD/CN average abundance ratios, with those having ratios above 2 and below 0.6 selected as being differential. Subsequently, 10 proteins meeting the criteria of an adjusted *p*‐value <0.05 were considered statistically significant. These proteins included: zinc finger protein 587B (ZNF587B), RNA helicase aquarius (AQR), C‐reactive protein (CRP), sialic acid‐binding Ig‐like lectin 16 (SIGLEC16), tropomyosin beta chain (TPM2), fibronectin (FN1), probable nonfunctional immunoglobulin kappa variable 1D‐37 (IGKV1D‐37), serum amyloid A‐1 protein (SAA1), hemoglobin subunit alpha (HBA1; HBA2), and myosin‐4 (MYH4). Based on their subcellular localization, we focused on nucleus proteins for their roles in gene regulation and selected secretory proteins for their involvement in various physiological processes. Finally, ZNF587B, AQR, CRP, FN1, and SAA1 were chosen for ROC analysis (Table 3). To assess their discriminatory ability, the cohort, consisting of individuals with AD and CN1 groups, was randomly divided into three datasets: the training dataset, which comprised 28 individuals (15 AD and 13 CN), the testing dataset, which included 9 individuals, and the validation dataset, which comprised 17 individuals. The proteins FN1, SAA1, CRP, ZNF587B, and AQR exhibited varying levels of performance, as indicated by their respective receiver operating characteristic (ROC) values, also known as the area under the curve (AUC). The highest AUC values were observed for FN1 with a score of 0.933, followed by SAA1 with 0.918, ZNF587B with 0.846, AQR with 0.810, and CRP with the AUC value of 0.692 (Fig. 2A).

**Table 3:** Characteristics of selected protein biomarkers obtained through mass spectrometry and their discriminatory calculation based on a training dataset.

Protein name/gene name	UniProt accession No.	Amino acids number	Molecular weight [kDa]	PSM^1^	Training ROC‐AUC^2^	Youden J Max^3^	Cutoff value (MS intensity)
Fibronectin/FN1	P02751	2477	272.2	4435	0.933	0.867	5,298,769,777
Serum amyloid A‐1 protein/SAA1	P0DJI8	122	13.5	333	0.918	0.8	50,429,916.51
C‐reactive protein/CRP	P02741	224	25	72	0.69	0.333	77,010,959.7
Zinc finger protein 587B/ZNF587B	E7ETH6	402	45.5	106	0.846	0.713	144,108,794.6
RNA helicase aquarius/AQR	O60306	1485	171.2	67	0.81	0.6	114,232,250.7

^1^ PSMs: peptide‐spectrum matchs, the number of PSMs is the total number of identified peptide spectra matched for the protein.

^2^ Training ROC‐AUC was calculated based on training dataset.

^3^ The maximum value of Youden's J statistic corresponds to the optimal cutoff point on the ROC curve.

**Figure 3:**
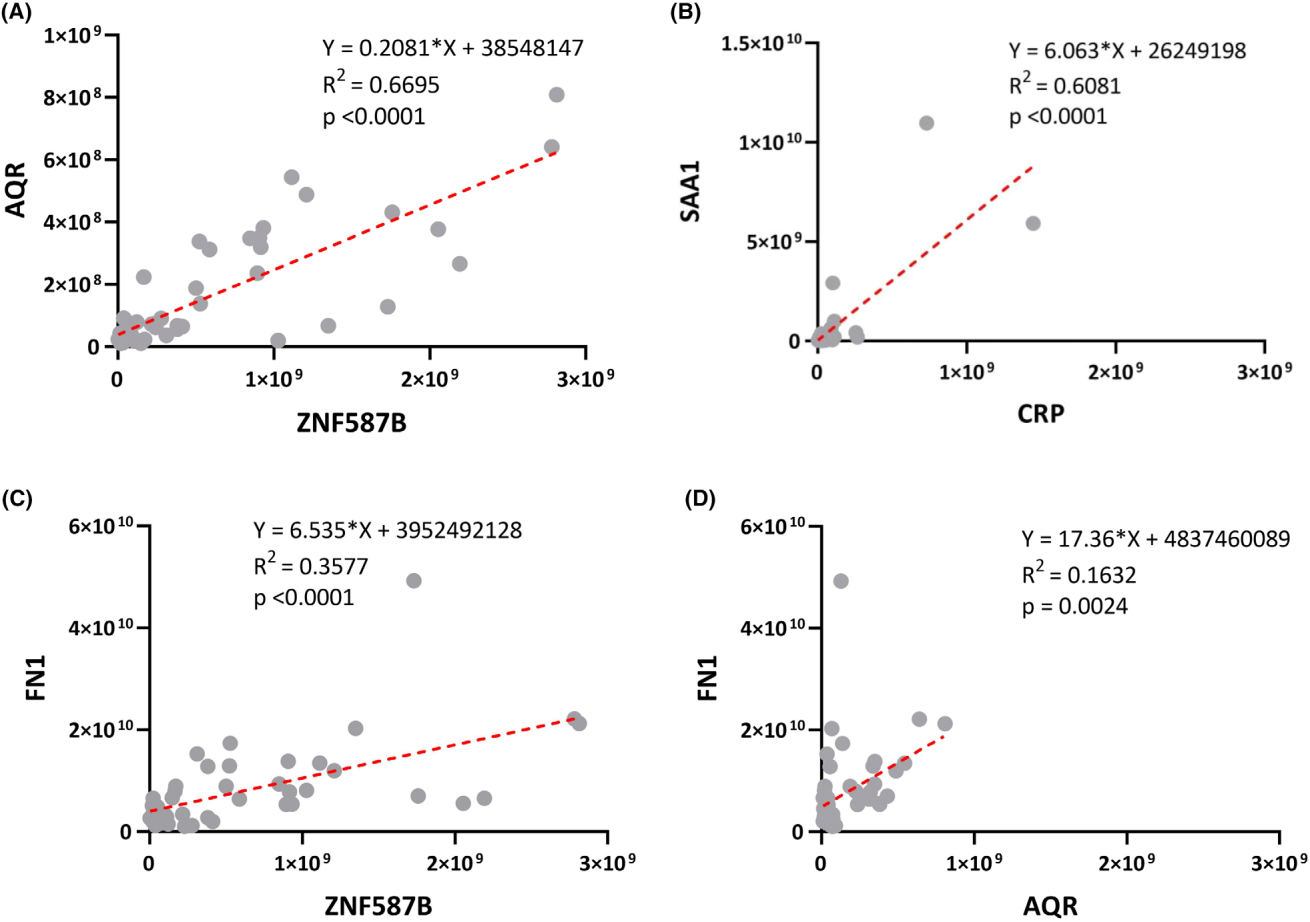
Linear regression analysis between each of two proteins. (A) AQR and ZNF587B, *r* ^2^ = 0.6695, *p* < 0.0001; (B) SAA1 and CRP, *r* ^2^ = 0.6081, *p* < 0.0001; (C) FN1 and ZNF587B, *r* ^2^ = 0.3577, *p* < 0.0001; (D) FN1 and AQR, *r* ^2^ = 0.1632, *p* < 0.01.

In accordance with the training model results, the evaluation of five protein biomarkers was conducted individually for testing dataset and validation dataset (Fig. 2B). The evaluation results of this analysis were represented using three metrics: true‐positive rate (TPR), true‐negative rate (TNR), and accuracy (ACC). In the case of testing dataset, the TPR values for ZNF587B, AQR, and FN1 were 83%, while SAA1 had a TPR of 50%, and CRP had a TPR of 33%. The TNR values were 100% for AQR, CRP, and SAA1, 67% for FN1, and 33% for ZNF587B. The corresponding ACC values were 67% for ZNF587B, AQR, and SAA1, 56% for CRP, and 78% for FN1 (Fig. 2C). In validation dataset, the TPR values were 63% for ZNF587B and FN1, 50% for SAA1 and AQR, and 25% for CRP. The TNR values were 100% for AQR and CRP, 89% for FN1 and SAA1, and 67% for ZNF587B. The ACC values were 76% for AQR and FN1, 71% for SAA1, and 65% for CRP (Fig. 2D). In addition, the linear correlation plots revealed strong correlations between ZNF587B and AQR (*R* ^2^ = 0.6695, *p* < 0.0001). Besides, significant correlations were observed between SAA1 and CRP (*R* ^2^ = 0.6081, *p* < 0.0001), ZNF587B and FN1 (*R* ^2^ = 0.3577, *p* < 0.0001), as well as between AQR and FN1 (*R* ^2^ = 0.1632, *p* = 0.0024) (Fig. 3).

### K‐Nearest Neighbors based diagnostic model for AD recognition

**Figure 4:**
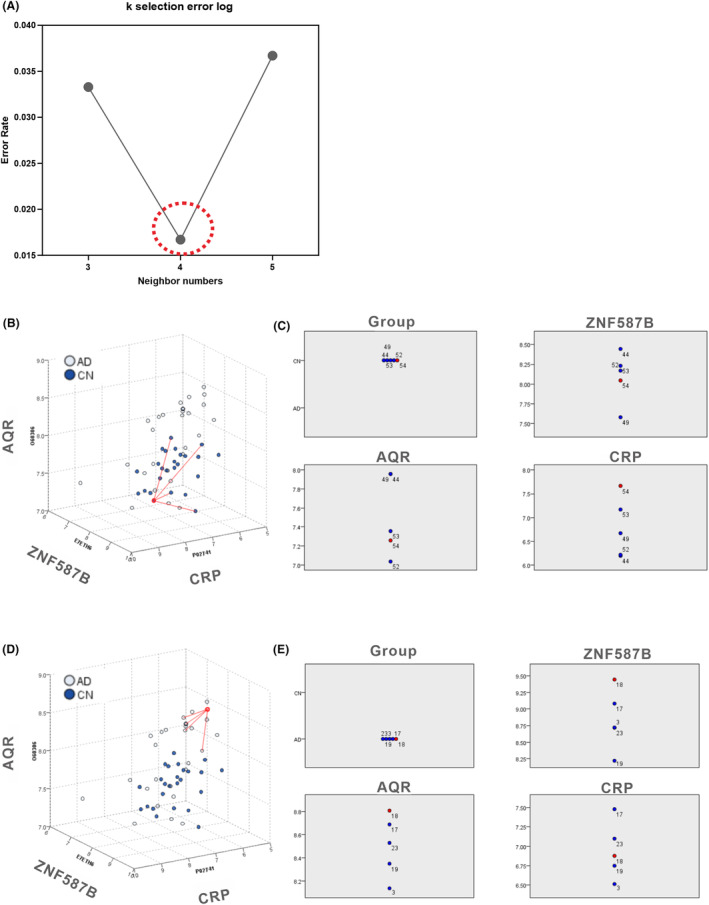
KNN analysis. (A) k selection error log, with the minimum error of 0.0167 at Number 4; (B) Selected CN focal case with its four neighbors in 3D model; (C) Selected CN focal case its four neighbors on peers chart; (D) Selected AD focal case with its four neighbors in 3D model; (E) Selected AD focal case its four neighbors on peers chart.

According to the KNN analysis, ZNF587B, AQR, and CRP were identified as the key components of the three‐dimensional model. To provide a concrete representation of the model, 3D models were created based on the X, Y, and Z coordinates of each sample. Specifically, X represents ZNF587B (UniProt ID: E7ETH6), Y represents CRP (P02741), and Z represents AQR (O60306). The focal case in either the AD or CN1 group, along with its four nearest neighbors, was chosen based on the values of ZNF587B, AQR, and CRP within the coordinates, as depicted in Figure 4B,D. The selected focal case in the feature space is displayed on the peers chart together with their four nearest neighbors in AD and CN1 group, respectively (Fig. 4C,E). The KNN model in AD diagnosis achieved AUC values of 0.941, 1.000, and 1.000 for the training, testing, and validation datasets, respectively (Table 4), indicating an excellent separation ability in the 3D diagnosis model.

**Table 4:** The accuracy and performance of a diagnostic model utilizing the K‐Nearest Neighbors (KNN) algorithm.

Group	Training dataset	Testing dataset	Validation dataset
Characteristics			
Model accuracy			
True	30 (96.77%)	8 (100%)	15 (100%)
False	1 (3.23%)	0 (0%)	0 (0%)
Performance measure			
AUC	0.941	1.000	1.000
Gini^1^	0.882	1.000	1.000

^1^ Gini is a metric that used to evaluate KNN classification models. The Gini coefficient ranges from 0 to 1, with 0 representing perfect equality (no discrimination) and 1 representing perfect inequality (perfect discrimination).

**Figure 5:**
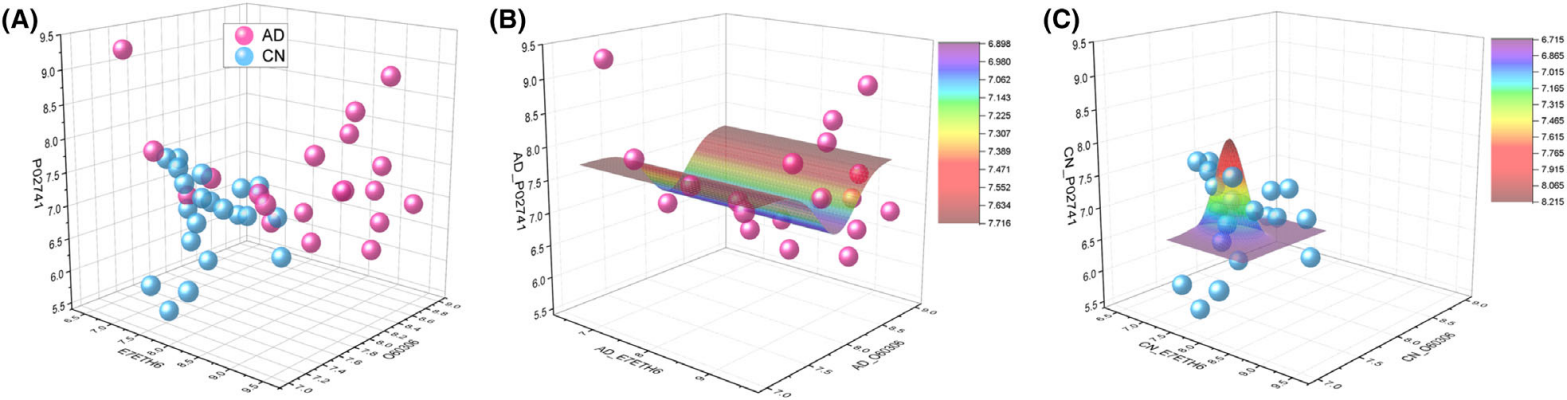
(A) Three‐dimensional model was built with ZNF587B, AQR and CRP, AD individuals were represented as red dots and CN individuals were represented as blue dots; (B) Nonlinear fit model generated a distinct wave‐shaped 3D model for AD group; (C) Nonlinear fit model generated a prominent hat‐shaped 3D model for CN group.

Furthermore, to illustrate the projection profiles of the AD and CN1 groups, the model was built with three features of ZNF587B, AQR, and CRP as depicted in Figure 5A. The nonlinear fit model generated a distinct wave‐shaped 3D model to represent the AD group, as shown in Figure 5B. The equation for this model was calculated as follows: *z* = *z*0 + A*exp(−0.5*((*x* − *xc*)/*w*1)^2^ − 0.5*((*y* − *yc*)/*w*2)^2^), *z*0 = 7.71447 ± 0.37699, *A* = −0.81556 ± 1.60552E7, *xc* = 1.53554E24 ± 2.60916E39, *w*1 = 1.01879E28 ± 1.57859E43, *yc* = 8.13763 ± 0.17634, *w*2 = 0.25117 ± 0.23375. Besides, the nonlinear fit resulted in a prominent hat‐shaped 3D model to represent the CN group, as depicted in Figure 5C. The equation for this model was calculated as *z* = *z*0 + *A* * exp(−0.5 * ((*x* − *xc*)/*w*1)^2^ − 0.5 * ((*y* − *yc*)/*w*2)^2^), *z*0 = 6.71895 ± 0.19783, *A* = 1.49591 ± 3.59014, *xc* = 7.86469 ± 0.14001, *w*1 = 0.22029 ± 0.19711, *yc* = 7.36064 ± 0.18199, *w2* = 0.09631 ± 0.22961.

### Serum metabolome profiling by LC–MS/MS

**Figure 6:**
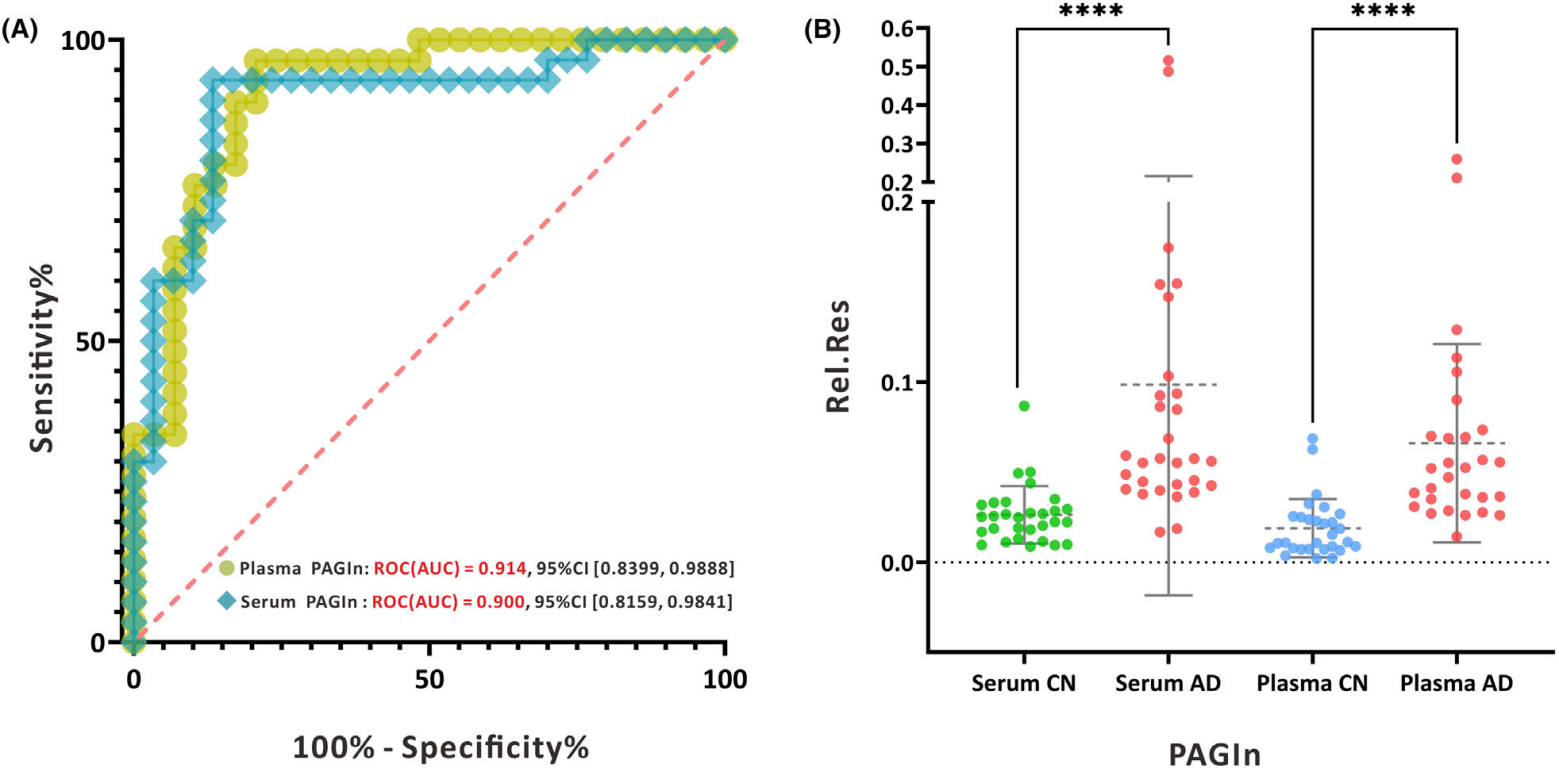
(A) ROC curves for PAGIn in serum and plasma samples both showed AUC values above 0.9; (B) Dot plots were used to visualize the upregulation of PAGIn in both serum and plasma samples from AD patients.

Serum metabolomics analysis was conducted on an independent group comprising 29 AD patients and 30 CN2 individuals. Mass spectrometry data were processed using Thermo Compound Discover 3.1 (CD 3.1, Thermo Scientific, USA), and all compound information is presented in Table S2. Data cleaning and semiquantitation were performed according to a protocol established in previous research.^15^ A total of 286 metabolites were screened, with 38 endogenous metabolites showing stable mass spectrometry intensities with coefficients of variation (CV%) below 30%, and these were selected for further analysis. Subsequently, receiver operating characteristic (ROC) analysis was conducted to identify potential metabolic features, as detailed in Table S3. Notably, serum phenylacetylglutamine (PAGln) exhibited an area under the ROC curve (ROC‐AUC) of 0.900, indicating excellent performance in distinguishing individuals with AD from CN individuals. This finding was consistent with our earlier results where plasma PAGln demonstrated an ROC‐AUC of 0.914. These outcomes suggest that both serum and plasma PAGIn exhibit strong sensitivity and specificity in discriminating AD from CN individuals (Fig. 6A). Furthermore, serum PAGIn levels were significantly higher in AD patients compared to controls (*p* < 0.001), consistently demonstrating an upregulation trend in PAGln compared to previous semiquantitative results in plasma (Fig. 6B).

## Discussion

Alzheimer's disease is the predominant cause of progressive dementia. Global burden of disease (GBD) 2019 reported that the number of people with dementia would increase to approximately 152.8 million cases in 2050.^20^ Currently, there is a lack of effective treatments or preventive measures available for this debilitating neurodegenerative disorder.^21^ Recent advances in multi‐omics approaches will facilitate the discovery of significant SASP molecules that may provide a better understanding of the underlying cellular and molecular mechanisms and the pathogenesis of AD. Consequently, these advances might enable the discovery of biomarkers suitable for improving the accuracy of AD diagnosis as well as novel therapeutics and preventive strategies.

In this work, we analyzed both proteomics and metabolomics features of AD patient's blood sample utilizing advanced mass spectrometers equipped with high‐resolution orbitrap mass analyzer system. Accurate identification of specific blood biomolecules has the potential to enhance the detection of cognitive decline features in future clinical diagnoses.

For proteomics analysis, plasma samples from AD patients (*N* = 29) and CN1 controls (*N* = 29) were subjected to analysis using nanoLC coupled to an Orbitrap Exploris 480 mass spectrometer. To maintain the integrity of low‐abundance proteins, we opted against depleting high‐abundance proteins during sample preparation. A total of 516 plasma proteins were identified through a combination of tandem mass spectrometry and a protein sequence database search. This approach represents a significant advancement of accurate protein identification in proteomic mass spectrometry, enhancing the potential for the discovery of proteomic markers in AD diagnostics.^13^, ^22^, ^23^ Traditional evaluation by ROC was used to screen those markers with potential power for AD discrimination.^24^, ^25^ Following the supervised learning workflow,^26^ we randomly divided the whole dataset into three groups for biomarkers training, testing, and validation.^18^, ^27^ The proteins AQR, ZNF587B, CRP, SAA1, and FN1 demonstrated promising performance in training dataset. However, their performance was not consistent in testing dataset and validation dataset. This incongruity could be attributed to the limited number of samples available in the dataset as well as the non‐normal distribution of MS intensities, which can exhibit either positive skewness or negative skewness. To enhance the model's fitness for diagnostic analysis, we applied a logarithmic transformation to normalize the skewed MS variables. Consequently, a machine learning‐based approach was employed to develop a model that can improve the accuracy and stability of AD prediction. The KNN algorithm was chosen for analyzing the proteomics data due to its suitability for high‐dimensional data analysis.^28^, ^29^ Notably, based on the KNN algorithm, we identified AQR, ZNF587B, and CRP as the three optimal features from a pool of 198 screened proteins.

Linear regression analysis showed a statistically significant relationship between AQR and ZNF587B, indicating an underlying pathophysiology in AD. AQR is a gene encoding intron‐binding protein, which is believed to be involved in RNA splicing. RNA helicases play a crucial role in unwinding RNA duplexes, facilitating interactions between RNAs and proteins. They have been linked to aging, lifespan regulation, and neurodegenerative diseases like amyotrophic lateral sclerosis and Alzheimer's disease.^30^ A recent study showed that AQR expression was significantly upregulated by 85.92% ± 3.75% in aged cells.^31^ Zinc finger proteins (ZNFs) are a diverse and abundant protein group with important biological functions. Changes in ZNFs have been associated with diseases such as neurodegeneration, skin disorders, and diabetes.^32^ Interestingly, gene expression analysis on peripheral blood specimens from major depressive disorder (MDD) patients and healthy controls revealed an upregulation of ZNF587 in individuals with MDD, in line with publicly available microarray data.^33^ Recent research has revealed the expression of ZNF417/587 in specific brain regions during development and adulthood is vital for regulating transposable elements in neurons derived from embryonic stem cells and brain organoids. This regulation indirectly impacts neuronal differentiation, neurotransmission profiles, and safeguards against the activation of neurotoxic retroviral proteins and an interferon‐like response.^34^

Besides, inflammation has long been thought to play a vital role in the pathophysiology of AD in older adults, whereby inflammation is related to further neurodegeneration and cognitive decline.^35^, ^36^, ^37^ C‐reactive protein, complement proteins, and serum amyloid A protein (SAA) are the principal acute phase proteins, mainly generated in the liver and released into the systemic circulation in response to inflammation.^38^, ^39^ CRP has the potential to affect blood brain barrier permeability and interacts with the central nervous system (CNS) as demonstrated by a strong correlation between CRP levels in plasma and cerebrospinal fluid (r > 0.85).^40^ Besides, CRP serves as an extensively investigated biomarker of inflammation and possesses the capability to activate the classical complement system, thereby inducing cell lysis and phagocytosis of cellular fragments.^41^, ^42^ An expanding body of evidence suggests that CRP, a well‐established marker of chronic inflammation, plays a pivotal role in the pathogenesis of AD.^43^

For metabolomics analysis, serum samples from AD patients (*N* = 29) and CN2 (*N* = 30) controls were processed following standard protocols, employing vanquish HPLC coupled with Q Exactive Plus mass spectrometer for metabolites profiling. A comprehensive database search led to the identification of 286 metabolites with confirmed compound names. Notably, the metabolite PAGIn exhibited the best performance, with an ROC‐AUC of 0.90, consistently aligning with previously reported findings.

PAGIn, a metabolite derived from the gut microbiota (GM), has been identified as significantly elevated in the plasma of patients with AD, and showed its strong correlation with Aβ42/Aβ40 in our previously work.^15^ In this study, involving different CN participants, we observed significantly higher serum PAGIn responses in AD patients. It was suggested AD patients exhibit distinct variations in gut microbial taxonomy compared to age‐matched controls, including decreased levels of Firmicutes and Actinobacteria, and increased species of Bacteroidetes.^44^ As GM is known to be involved in the shaping of the immune system during early life. The identification of intestinal dysbiosis in patients with neurological disorders has highlighted the importance of gut–brain communication, and yet the question regarding the identity of the components responsible for this crosstalk remains open. However, the connections between systemic metabolic abnormalities and the pathogenesis of AD are still unclear.

Recent studies also suggested a role for the GM in the regulation of inflammation by influencing differentiation of inflammatory cell types, cytokine production and hematopoiesis.^45^, ^46^ A leaky gut and alterations in GM composition can both lead to leakage of endotoxins into the circulation that promotes systemic inflammation and to the development of obesity or related metabolic diseases.^47^ Besides, extensive research has established connections between various microbial factors and the pathogenesis of AD.^48^, ^49^ Furthermore, compelling evidence suggests a correlation between changes in GM composition and AD.^50^, ^51^, ^52^ Research conducted with transgenic mouse models devoid of GM has showcased diminished amyloid buildup within the cerebral region, implying the impact of microbial amyloids on neuroinflammation and the levels of β‐amyloid peptides via the modulation of reactive gliosis in the brain.^53^ As we found that GM alterations in AD potentially induce peripheral and central immunological changes via the release of microbial metabolites, we propose that modulating their composition may alter ongoing inflammation and could therefore be a promising future strategy to fight progression of AD.

Our results found AQR, ZNF587B, CRP, and PAGIn have been accumulated in AD patients as well as been reported associated with neurodegenerative diseases, while their relationship with AD has not been thoroughly examined. Cellular senescence, an endogenous mechanism of aging, has recently been linked to dysbiosis, wherein the GM exerts its influence on cellular senescence through the release of microbial metabolites.^54^ Besides, accumulation of senescent cells in AD has been linked to an overexpression of the SASP, characterized by proinflammatory cytokines, chemokines, growth factors, proteases, metabolites, lipids and extracellular matrix components. It is likely that age‐related alterations in the GM facilitate inflammatory processes that may contribute to the neuro‐inflammation in AD. However, proving that there is an infective cause to the neuro‐inflammation and neurodegeneration seen in patients with AD is logistically and ethically challenging in humans. Thus, the underlying pathophysiology of these AD‐associated molecules is worth to be studied in the future.

Nevertheless, this study has several limitations. First, the small sample size of our study represented a major constraint, particularly when employing machine learning techniques for analyzing extensive datasets. Besides, our results should be considered in light of potential confounders such as APOE genotype, gender, and body mass index, which were not taken into consideration. Additionally, our findings warrant further investigations, special at the tissue level, to elucidate the precise molecules changes within specific pathways and network contexts. This shall provide a more comprehensive understanding of the underlying mechanisms.

## Conclusions

In this study, we performed a comprehensive multi‐omics analysis to investigate the senescence‐associated secretory phenotypes (SASPs) associated with Alzheimer's disease (AD). Our results identified three proteins, namely AQR, ZNF587B, and CRP, as well as a metabolite called PAGIn, as SASPs linked to AD. The presence of these markers suggests their potential usefulness as informative diagnostic indicators for AD. Utilizing mass spectrometry‐based analysis, we successfully profiled the blood proteome and metabolome of AD patients, allowing for the simultaneous measurement of multiple biomarkers. This approach significantly improved the efficiency of biomarker discovery for accurate diagnosis. Furthermore, we applied a machine learning algorithm, KNN, to identify highly correlated biomarkers and construct an AD diagnostic model. This machine learning approach demonstrated specificity and accuracy in establishing a comprehensive diagnostic framework. Nevertheless, further investigations are crucial to assess the stability and reliability of these SASPs, which have also demonstrated diagnostic potential for AD.

## Funding Information

This work was supported by the National Key R&D Program of China (No. 2021YFC2100201).

## Author Contributions

Conceptualization: X Dong and L Zhao; investigation: JZ Yang and YG Zhou; methodology and software: JZ Yang and TJ Wang; formal analysis and data curation: JZ Yang; supervision and validation: Q Zhang and S Wu; writing—original draft preparation: JZ Yang, YG Zhou, and TJ Wang; writing—editing: N Li, YF Chao, and SY Gao; Writing—review: X Dong and L Zhao; funding acquisition: X Dong. All authors approved the final version of this manuscript.

## Conflict of Interest

All authors declare to have no competing interests with the content of this article.

## Consent for Publication

Not applicable.

## Supporting information


Figure S1.

Table S1.

Table S2.

Table S3.

Figure S1 Caption.

## Data Availability

All the data generated and analyzed in this work are included in the manuscript and the supplementary materials.

## References

[1] 2023 Alzheimer's disease facts and figures. Alzheimers Dement. 2023;19:1598‐1695. 36918389 10.1002/alz.13016

[2] Salminen A , Ojala J , Kaarniranta K , Haapasalo A , Hiltunen M , Soininen H . Astrocytes in the aging brain express characteristics of senescence‐associated secretory phenotype. Eur J Neurosci. 2011;34:3‐11. 21649759 10.1111/j.1460-9568.2011.07738.x

[3] Efthymiou AG , Goate AM . Late onset Alzheimer's disease genetics implicates microglial pathways in disease risk. Mol Neurodegener. 2017;12:43. 28549481 10.1186/s13024-017-0184-x PMC5446752

[4] Guerrero A , De Strooper B , Arancibia‐Carcamo IL . Cellular senescence at the crossroads of inflammation and Alzheimer's disease. Trends Neurosci. 2021;44:714‐727. 34366147 10.1016/j.tins.2021.06.007

[5] Watanabe S , Kawamoto S , Ohtani N , Hara E . Impact of senescence‐associated secretory phenotype and its potential as a therapeutic target for senescence‐associated diseases. Cancer Sci. 2017;108:563‐569. 28165648 10.1111/cas.13184 PMC5406532

[6] Basisty N , Kale A , Patel S , Campisi J , Schilling B . The power of proteomics to monitor senescence‐associated secretory phenotypes and beyond: toward clinical applications. Expert Rev Proteomics. 2020;17:297‐308. 32425074 10.1080/14789450.2020.1766976 PMC7416420

[7] Li X , Wang W , Chen J . Recent progress in mass spectrometry proteomics for biomedical research. Sci China Life Sci. 2017;60:1093‐1113. 29039124 10.1007/s11427-017-9175-2

[8] Jacob M , Lopata AL , Dasouki M , Abdel Rahman AM . Metabolomics toward personalized medicine. Mass Spectrom Rev. 2019;38:221‐238. 29073341 10.1002/mas.21548

[9] Kudryashova KS , Burka K , Kulaga AY , Vorobyeva NS , Kennedy BK . Aging biomarkers: from functional tests to multi‐omics approaches. Proteomics. 2020;20:e1900408. 32084299 10.1002/pmic.201900408

[10] Duong VA , Lee H . Bottom‐up proteomics: advancements in sample preparation. Int J Mol Sci. 2023;24:5350. 36982423 10.3390/ijms24065350 PMC10049050

[11] Horgusluoglu E , Neff R , Song WM , et al. Integrative metabolomics‐genomics approach reveals key metabolic pathways and regulators of Alzheimer's disease. Alzheimers Dement. 2022;18:1260‐1278. 34757660 10.1002/alz.12468 PMC9085975

[12] Zhang Z , Wu S , Stenoien DL , Pasa‐Tolic L . High‐throughput proteomics. Annu Rev Anal Chem (Palo Alto, Calif). 2014;7:427‐454. 25014346 10.1146/annurev-anchem-071213-020216

[13] Bai B , Vanderwall D , Li Y , et al. Proteomic landscape of Alzheimer's disease: novel insights into pathogenesis and biomarker discovery. Mol Neurodegener. 2021;16:55. 34384464 10.1186/s13024-021-00474-z PMC8359598

[14] Varma VR , Oommen AM , Varma S , et al. Brain and blood metabolite signatures of pathology and progression in Alzheimer disease: a targeted metabolomics study. PLoS Med. 2018;15:e1002482. 29370177 10.1371/journal.pmed.1002482 PMC5784884

[15] Yang J , Wu S , Yang J , Zhang Q , Dong X . Amyloid beta‐correlated plasma metabolite dysregulation in Alzheimer's disease: an untargeted metabolism exploration using high‐resolution mass spectrometry toward future clinical diagnosis. Front Aging Neurosci. 2023;15:1189659. 37455936 10.3389/fnagi.2023.1189659 PMC10338932

[16] Barla A , Jurman G , Riccadonna S , Merler S , Chierici M , Furlanello C . Machine learning methods for predictive proteomics. Brief Bioinform. 2008;9:119‐128. 18310105 10.1093/bib/bbn008

[17] Elgammal YM , Zahran MA , Abdelsalam MM . A new strategy for the early detection of alzheimer disease stages using multifractal geometry analysis based on K‐Nearest Neighbor algorithm. Sci Rep. 2022;12:22381. 36572791 10.1038/s41598-022-26958-6 PMC9792538

[18] Shen B , Yi X , Sun Y , et al. Proteomic and metabolomic characterization of COVID‐19 patient sera. Cell. 2020;182:59‐72.e15. 32492406 10.1016/j.cell.2020.05.032 PMC7254001

[19] Di Guida R , Engel J , Allwood JW , et al. Non‐targeted UHPLC‐MS metabolomic data processing methods: a comparative investigation of normalisation, missing value imputation, transformation and scaling. Metabolomics. 2016;12:93. 27123000 10.1007/s11306-016-1030-9 PMC4831991

[20] Collaborators GBDDF . Estimation of the global prevalence of dementia in 2019 and forecasted prevalence in 2050: an analysis for the Global Burden of Disease Study 2019. Lancet Public Health. 2022;7:e105‐e125. 34998485 10.1016/S2468-2667(21)00249-8 PMC8810394

[21] Scheltens P , De Strooper B , Kivipelto M , et al. Alzheimer's disease. Lancet. 2021;397:1577‐1590. 33667416 10.1016/S0140-6736(20)32205-4 PMC8354300

[22] Dayon L , Cominetti O , Affolter M . Proteomics of human biological fluids for biomarker discoveries: technical advances and recent applications. Expert Rev Proteomics. 2022;19:131‐151. 35466824 10.1080/14789450.2022.2070477

[23] Xie F , Smith RD , Shen Y . Advanced proteomic liquid chromatography. J Chromatogr A. 2012;1261:78‐90. 22840822 10.1016/j.chroma.2012.06.098 PMC3463731

[24] Lasko TA , Bhagwat JG , Zou KH , Ohno‐Machado L . The use of receiver operating characteristic curves in biomedical informatics. J Biomed Inform. 2005;38:404‐415. 16198999 10.1016/j.jbi.2005.02.008

[25] Alemayehu D , Zou KH . Applications of ROC analysis in medical research: recent developments and future directions. Acad Radiol. 2012;19:1457‐1464. 23122565 10.1016/j.acra.2012.09.006

[26] Jiang T , Gradus JL , Rosellini AJ . Supervised machine learning: a brief primer. Behav Ther. 2020;51:675‐687. 32800297 10.1016/j.beth.2020.05.002 PMC7431677

[27] Deo RC . Machine learning in medicine. Circulation. 2015;132:1920‐1930. 26572668 10.1161/CIRCULATIONAHA.115.001593 PMC5831252

[28] Alkady W , ElBahnasy K , Gad W . A diagnostic model for COVID‐19 based on proteomics analysis. Comput Biol Med. 2023;162:107109. 37276752 10.1016/j.compbiomed.2023.107109 PMC10232940

[29] Guldberg SM , Okholm TLH , McCarthy EE , Spitzer MH . Computational methods for single‐cell proteomics. Annu Rev Biomed Data Sci. 2023;6:47‐71. 37040735 10.1146/annurev-biodatasci-020422-050255 PMC10621466

[30] Steimer L , Klostermeier D . RNA helicases in infection and disease. RNA Biol. 2012;9:751‐771. 22699555 10.4161/rna.20090

[31] Wan Y , Liu Z , Wu A , et al. Hyperglycemia promotes endothelial cell senescence through AQR/PLAU signaling Axis. Int J Mol Sci. 2022;23:2879. 35270021 10.3390/ijms23052879 PMC8911151

[32] Cassandri M , Smirnov A , Novelli F , et al. Zinc‐finger proteins in health and disease. Cell Death Dis. 2017;3:17071. 10.1038/cddiscovery.2017.71 PMC5683310 29152378

[33] Woo HI , Lim SW , Myung W , Kim DK , Lee SY . Differentially expressed genes related to major depressive disorder and antidepressant response: genome‐wide gene expression analysis. Exp Mol Med. 2018;50:1‐11. 10.1038/s12276-018-0123-0 PMC6076250 30076325

[34] Turelli P , Playfoot C , Grun D , et al. Primate‐restricted KRAB zinc finger proteins and target retrotransposons control gene expression in human neurons. Sci Adv. 2020;6:eaba3200. 32923624 10.1126/sciadv.aba3200 PMC7455193

[35] Nordengen K , Kirsebom BE , Henjum K , et al. Glial activation and inflammation along the Alzheimer's disease continuum. J Neuroinflammation. 2019;16:46. 30791945 10.1186/s12974-019-1399-2 PMC6383268

[36] McQuade A , Blurton‐Jones M . Microglia in Alzheimer's disease: exploring how genetics and phenotype influence risk. J Mol Biol. 2019;431:1805‐1817. 30738892 10.1016/j.jmb.2019.01.045 PMC6475606

[37] Ferguson SA , Varma V , Sloper D , Panos JJ , Sarkar S . Increased inflammation in BA21 brain tissue from African Americans with Alzheimer's disease. Metab Brain Dis. 2020;35:121‐133. 31823110 10.1007/s11011-019-00512-2

[38] Gabay C , Kushner I . Acute‐phase proteins and other systemic responses to inflammation. N Engl J Med. 1999;340:448‐454. 9971870 10.1056/NEJM199902113400607

[39] Facci L , Barbierato M , Zusso M , Skaper SD , Giusti P . Serum amyloid a primes microglia for ATP‐dependent interleukin‐1beta release. J Neuroinflammation. 2018;15:164. 29803222 10.1186/s12974-018-1205-6 PMC5970445

[40] Felger JC , Haroon E , Patel TA , et al. What does plasma CRP tell us about peripheral and central inflammation in depression? Mol Psychiatry. 2020;25:1301‐1311. 29895893 10.1038/s41380-018-0096-3 PMC6291384

[41] Koyama A , O'Brien J , Weuve J , Blacker D , Metti AL , Yaffe K . The role of peripheral inflammatory markers in dementia and Alzheimer's disease: a meta‐analysis. J Gerontol A Biol Sci Med Sci. 2013;68:433‐440. 22982688 10.1093/gerona/gls187 PMC3693673

[42] McGeer EG , McGeer PL . The importance of inflammatory mechanisms in Alzheimer disease. Exp Gerontol. 1998;33:371‐378. 9762518 10.1016/s0531-5565(98)00013-8

[43] Natale G , Clouston SAP , Smith DM . Elevated C‐reactive protein in Alzheimer's disease without depression in older adults: findings from the health and retirement study. J Gerontol A Biol Sci Med Sci. 2022;77:673‐682. 34671810 10.1093/gerona/glab282 PMC8974321

[44] Roth W , Zadeh K , Vekariya R , Ge Y , Mohamadzadeh M . Tryptophan metabolism and gut‐brain homeostasis. Int J Mol Sci. 2021;22:2973. 33804088 10.3390/ijms22062973 PMC8000752

[45] Schirmer M , Smeekens SP , Vlamakis H , et al. Linking the human gut microbiome to inflammatory cytokine production capacity. Cell. 2016;167:1125‐1136.e8. 27814509 10.1016/j.cell.2016.10.020 PMC5131922

[46] Geva‐Zatorsky N , Sefik E , Kua L , et al. Mining the human gut microbiota for immunomodulatory organisms. Cell. 2017;168:928‐943.e11. 28215708 10.1016/j.cell.2017.01.022 PMC7774263

[47] Cani PD , Osto M , Geurts L , Everard A . Involvement of gut microbiota in the development of low‐grade inflammation and type 2 diabetes associated with obesity. Gut Microbes. 2012;3:279‐288. 22572877 10.4161/gmic.19625 PMC3463487

[48] Geuking MB , Cahenzli J , Lawson MA , et al. Intestinal bacterial colonization induces mutualistic regulatory T cell responses. Immunity. 2011;34:794‐806. 21596591 10.1016/j.immuni.2011.03.021

[49] Atarashi K , Tanoue T , Shima T , et al. Induction of colonic regulatory T cells by indigenous clostridium species. Science. 2011;331:337‐341. 21205640 10.1126/science.1198469 PMC3969237

[50] Zhuang Z , Yang R , Wang W , Qi L , Huang T . Associations between gut microbiota and Alzheimer's disease, major depressive disorder, and schizophrenia. J Neuroinflammation. 2020;17:288. 33008395 10.1186/s12974-020-01961-8 PMC7532639

[51] Doifode T , Giridharan VV , Generoso JS , et al. The impact of the microbiota‐gut‐brain axis on Alzheimer's disease pathophysiology. Pharmacol Res. 2021;164:105314. 33246175 10.1016/j.phrs.2020.105314

[52] Hu X , Wang T , Jin F . Alzheimer's disease and gut microbiota. Sci China Life Sci. 2016;59:1006‐1023. 27566465 10.1007/s11427-016-5083-9

[53] Harach T , Marungruang N , Duthilleul N , et al. Reduction of Abeta amyloid pathology in APPPS1 transgenic mice in the absence of gut microbiota. Sci Rep. 2017;7:41802. 28176819 10.1038/srep41802 PMC5297247

[54] Boyajian JL , Ghebretatios M , Schaly S , Islam P , Prakash S . Microbiome and human aging: probiotic and prebiotic potentials in longevity, skin health and cellular senescence. Nutrients. 2021;13:4550. 34960102 10.3390/nu13124550 PMC8705837

