# Supplementary figures and images for: A multi‐omics study to monitor senescence‐associated secretory phenotypes of Alzheimer's disease
Source: Ann Clin Transl Neurol. 2024 Apr 11;11(5):1310–24. doi: 10.1002/acn3.52047 (PMC11093245; doi:10.1002/acn3.52047)

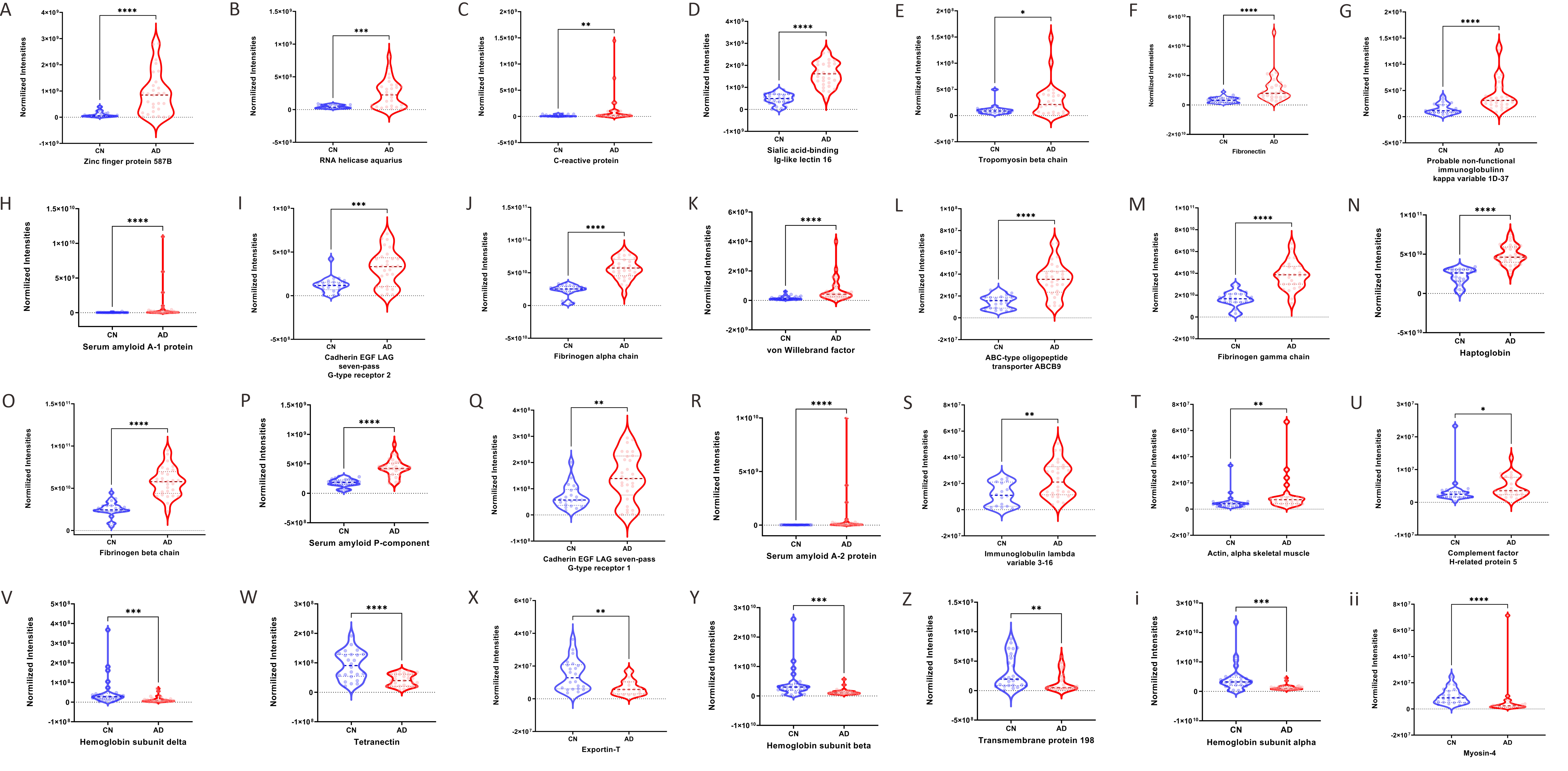

Supplement: Supplementary file 1 — Figure S1. [file ACN3-11-1310-s002.png]
