# Supplementary material for: A multi‐omics study to monitor senescence‐associated secretory phenotypes of Alzheimer's disease
Source: Ann Clin Transl Neurol. 2024 Apr 11;11(5):1310–24. doi: 10.1002/acn3.52047 (PMC11093245; doi:10.1002/acn3.52047)
Supplement: Supplementary file 4 — Table S3. [file ACN3-11-1310-s004.docx]

**Supplementary Table 3.** ROC analysis of serum metabolites in individuals with AD and CN2 individuals

| **No.** | **Compound name** | **ROC-AUC** | **Standard deviation** | **p value** | **Lower limits of 95% confidence interval** | **Upper limits of 95% confidence interval** |
| --- | --- | --- | --- | --- | --- | --- |
| 1 | Phenylacetylglutamine | 0.900 | 0.043 | 0 | 0.816 | 0.984 |
| 2 | Creatine | 0.792 | 0.058 | 0 | 0.678 | 0.907 |
| 3 | DL-Stachydrine | 0.773 | 0.061 | 0 | 0.653 | 0.894 |
| 4 | DL-Glutamine | 0.702 | 0.071 | 0.007 | 0.564 | 0.841 |
| 5 | Propionylcarnitine | 0.698 | 0.068 | 0.008 | 0.564 | 0.831 |
| 6 | Indole-3-acetic acid | 0.686 | 0.073 | 0.014 | 0.543 | 0.828 |
| 7 | Proline | 0.684 | 0.069 | 0.014 | 0.549 | 0.82 |
| 8 | Prolylleucine | 0.678 | 0.073 | 0.018 | 0.534 | 0.821 |
| 9 | Glycerophospho-N-palmitoyl ethanolamine | 0.677 | 0.07 | 0.019 | 0.54 | 0.813 |
| 10 | D-Carnitine | 0.671 | 0.07 | 0.023 | 0.533 | 0.809 |
| 11 | L-(+)-Arginine | 0.639 | 0.072 | 0.065 | 0.497 | 0.781 |
| 12 | Acetyl-β-methylcholine | 0.590 | 0.074 | 0.231 | 0.444 | 0.736 |
| 13 | Leucine | 0.571 | 0.075 | 0.344 | 0.424 | 0.718 |
| 14 | 4-Hydroxybenzaldehyde | 0.547 | 0.077 | 0.535 | 0.397 | 0.697 |
| 15 | Palmitoylcarnitine | 0.529 | 0.076 | 0.701 | 0.379 | 0.679 |
| 16 | Oleamide | 0.521 | 0.076 | 0.779 | 0.373 | 0.669 |
| 17 | L-Tyrosine | 0.499 | 0.078 | 0.988 | 0.345 | 0.652 |
| 18 | L-Histidine | 0.498 | 0.076 | 0.976 | 0.348 | 0.648 |
| 19 | δ-Valerolactam | 0.491 | 0.078 | 0.906 | 0.338 | 0.644 |
| 20 | L-Isoleucine | 0.464 | 0.075 | 0.636 | 0.317 | 0.612 |
| 21 | L-(-)-Methionine | 0.462 | 0.075 | 0.615 | 0.314 | 0.61 |
| 22 | Choline | 0.429 | 0.075 | 0.344 | 0.282 | 0.575 |
| 23 | Acetylcholine | 0.419 | 0.075 | 0.28 | 0.273 | 0.565 |
| 24 | Diisobutylphthalate | 0.416 | 0.076 | 0.261 | 0.267 | 0.564 |
| 25 | 2,2,6,6-Tetramethyl-4-piperidinol | 0.400 | 0.074 | 0.183 | 0.254 | 0.546 |
| 26 | D-Erythro-sphingosine 1-phosphate | 0.398 | 0.077 | 0.174 | 0.247 | 0.549 |
| 27 | Buprenorphine | 0.384 | 0.074 | 0.124 | 0.239 | 0.53 |
| 28 | Betaine | 0.362 | 0.073 | 0.067 | 0.218 | 0.506 |
| 29 | Uric acid | 0.359 | 0.073 | 0.06 | 0.215 | 0.502 |
| 30 | Acetyl-L-carnitine | 0.341 | 0.072 | 0.035 | 0.2 | 0.482 |
| 31 | Valine | 0.339 | 0.071 | 0.032 | 0.2 | 0.478 |
| 32 | Hypoxanthine | 0.331 | 0.073 | 0.025 | 0.188 | 0.474 |
| 33 | Lactamide | 0.327 | 0.071 | 0.021 | 0.187 | 0.467 |
| 34 | 2-Ethoxy-2'-ethyloxanilide | 0.278 | 0.067 | 0.003 | 0.147 | 0.409 |
| 35 | Isoleucine | 0.276 | 0.067 | 0.003 | 0.144 | 0.407 |
| 36 | L-Pyroglutamic acid | 0.253 | 0.063 | 0.001 | 0.129 | 0.378 |
| 37 | Bilirubin | 0.252 | 0.065 | 0.001 | 0.126 | 0.379 |
| 38 | Decanoylcarnitine | 0.241 | 0.065 | 0.001 | 0.114 | 0.368 |
