# Supplementary material for: A multi‐omics study to monitor senescence‐associated secretory phenotypes of Alzheimer's disease
Source: Ann Clin Transl Neurol. 2024 Apr 11;11(5):1310–24. doi: 10.1002/acn3.52047 (PMC11093245; doi:10.1002/acn3.52047)
Supplement: Supplementary file 5 — Figure S1 Caption. [file ACN3-11-1310-s003.docx]

**Figure S1.** Boxplots were constructed to display the differential expression of 28 plasma proteins in individuals with AD and CN.
